# Gendered Body Mass and Life Satisfaction Among Youth in Three Western European Immigrant-Receiving Countries

**DOI:** 10.3389/fsoc.2021.695374

**Published:** 2021-12-10

**Authors:** Jing Shen, Irena Kogan

**Affiliations:** Mannheim Centre for European Social Research, A 5, 6, Building A, University of Mannheim, Mannheim, Germany

**Keywords:** life satisfaction, body mass index, Western Europe, social relations, self-esteem, mental stability, ethno-racial diversity, adolescents

## Abstract

In this study we aim to show distinctive patterns of the association between body weight and life satisfaction for adolescent boys and girls, respectively. We understand such patterns by bringing multiple mediating factors into one theoretical framework centred on normative perceptions. By drawing data from the first wave of the CILS4EU that captures 14–15-year-olds in Germany, the Netherlands and Sweden, findings show that psychological factors, indicated by self-esteem and mental state, explain the association between BMI and life dissatisfaction substantially, for both boys and girls. Relationships with parents (particularly among boys) and relationships with peers (particularly among girls) also play significant roles. Moreover, the association between being underweight and life satisfaction among girls varies across ethno-racial groups. Girls originating from Eastern Europe have a tendency to gain more life satisfaction when being underweight, whereas girls rooted in Sub-Saharan African and Caribbean countries display consistently low levels of life satisfaction when being underweight.

## Introduction

Adolescence is an important stage of an individual’s development and is marked by transitions in young people’s lives in multiple dimensions, including the completion of compulsory education, entry into first romantic relationships, formation of attitudes, and consolidation of religious views. The extent to which adolescents master these transitions successfully might have an impact on their subjective well-being or life satisfaction, not only in the adolescent stage but also throughout their lives (Olsson et al., 2013; Switek and Easterlin, 2018).

In this period, appearance gains particular importance in youth’s life satisfaction. Due to rapid physiological transformations, the lack of satisfaction with one’s body or looks is not uncommon among adolescents. During puberty, while boys tend to experience rapid growth which results in their bodies being tall but often overly lean and unmuscular, girls are more prone to gaining body fat. Such changes might cause dissatisfaction and even distress among adolescents, if they self-perceive as failing to meet the mainstream beauty standards in their societies.

Existing studies from American and other predominantly non-European contexts have examined the relationship between body mass and life satisfaction among adolescents, and largely demonstrated distinctive patterns for girls and boys (Pingitore et al., 1997; Vincent and McCabe, 2000; Cohane and Pope, 2001; Mäkinen et al., 2012; Agam et al., 2015). However, explanations for those patterns are inconclusive. This study thus aims to fill the research gap by bringing multiple mediating factors into one explanatory model to understand how teenage boys and girls perceive and feel about their body weight in different ways.

From the socio-psychological perspective, life satisfaction is a subjective evaluation process, in which individuals compare their (perceived) situations with their hopes or expectations of how the situation should be—namely an ideal situation (Campbell et al., 1976; Shin and Johnson, 1978; see also Michalos, 1985 for an extensive overview). Whereas factual situations can largely be captured by individuals’ current state and circumstances, one’s perception of an ideal situation involves far more complicated mechanisms. In terms of the relationship between body weight and life satisfaction, we argue that both external and internal factors play important roles in shaping youth’s self-perceptions; namely, how close they are to the ideal body weight. Externally, we will examine social influences by focusing on a teenager’s relationships with parents, teachers and peers. Internally, we will examine psychological factors, indicated by self-esteem and mental state. By paying particular attention to youth with migration backgrounds, we will further examine whether or not and how teen boys’ and girls’ subjective evaluations of body weight vary across ethno-racial groups. Using data from three European countries—Germany, the Netherlands and Sweden, this study will show whether gendered patterns of the association between body weight and life satisfaction, established upon empirical evidence in America and other non-European contexts, would hold true in the context of Western European countries.

## Theoretical Framework

### Gendered Normative Perceptions of Body Weight

The perspective of *normative expectations* (Elster, 1989; Coleman, 1990; Hechter and Opp, 2001) indicates that life satisfaction is elevated when one’s own circumstances resemble those considered appropriate in a given society. Regarding body weight, the ideal state and the ways of achieving it are commonly transmitted by social media and the entertainment industry in modern societies. Through those channels, cultural norms defining an idealized appearance and body weight are formed, disseminated, and further reinforced. This implies that normative perceptions of body weight are context-specific. For example, in the Western world, and particularly among higher socioeconomic strata, the perceived perfect body is a slender one (Swami et al., 2010), whereas being obese or overweight might be less psychologically burdensome in societies where heavy body weight is more acceptable (Wadsworth and Pendergast, 2014).

Context-specific norms about body weight/size drive the importance of appearance for success and the fulfilment of one’s own worth through social influences, which in turn, has direct consequences on life satisfaction. This is because one’s self-perception of worthiness, sense of dignity and feeling of happiness are often gained through interactions with others, and negative feedback from others has proven being associated with low levels of subjective well-being (Vincent and McCabe, 2000). Regarding body awareness, adolescents often form their perceptions based on feedback from the immediate environment, and particularly those influential in their daily lives. As argued by Vincent and McCabe (2000), being teased or criticised of one’s body weight/size by *family members* internalizes an ideal body image and is a predictor of body dissatisfaction and eating disorder. Summarizing research on within-family experiences among overweight children, Forste and Moore (2012) point out that children beyond the normal weight range are likely to experience more negative within-family interactions. They are under constant pressure to diet, have stressful mealtimes and frequent conflicts with other family members. *Friends and peers* are a second salient source of social influences, as a large proportion of adolescents, particularly girls, are frequently engaged in discussions and even direct comparisons about body/physical looks with their peers. According to Dohnt and Tiggemann (2005), weight-related teasing among peers reinforces idealized body images as well as dieting behaviour among adolescents, and directly triggers youth’s body dissatisfaction, especially when their body weight/size falls outside the “acceptable” range (Forste and Moore, 2012). As a result, underweight and overweight adolescents tend to have lower levels of subjective well-being than their normal-weight counterparts, due to different social encounters they experience (Forste and Moore, 2012). *Teachers* constitute the last indispensable source of social influences in youth’s daily lives. Overweight youngsters have a higher risk of being stigmatized and even discriminated against by their teachers at school, which may further decrease the already low levels of life satisfaction among them (Sabia, 2007).

It must be pointed out that the formation of perceptions of an ideal body image is a gendered process (Vincent and McCabe, 2000; Cohane and Pope, 2001; Mäkinen et al., 2012; Agam et al., 2015). Boys tend to feel dissatisfied with their bodies when they are either below or above the normal weight, namely, an inverse U-shaped relationship between body weight, indicated by the Body Mass Index (BMI hereafter) and subjective well-being is observed (Mäkinen et al., 2012; Linna et al., 2013). By contrast, girls’ body dissatisfaction increases with their body weight (Pingitore et al., 1997; Mäkinen et al., 2012). Sources of these gender differences can largely be attributed to normative perceptions. As Frederick et al. (2007) stated, an ideal male body is a big, masculine one, which consequently gives rise to boys’ preference for a pronounced body mass as it is considered a sign of muscularity. An ideal female body, particularly within the Western context, is a slender one. The difference between the ideal of thinness for a female body and the ideal of muscularity for a male body naturally leads to gender-specific associations between BMI and life satisfaction, meaning that such relationships must be examined for boys and girls separately.

### Psychological Mediators in the Internalization of Normative Perceptions

The above section has highlighted the importance of societal contexts and social interactions in the formation of normative perceptions. Nevertheless, the extent to which those two aspects influence youth’s self-perceptions of their body weight/size depends on how they internalize influences of cultural norms, especially imposed by the immediate environment. Such an internalization process is highly contingent on *psychological factors*. For example, youth’s self-esteem, defined as the extent to which they like and accept themselves, is known to predict the ways by which they respond to challenges and behave in critical situations. Individuals with high self-esteem are more likely to cope better with stress and feel good about their lives (Schachter et al., 2014). Those with low self-esteem, on the contrary, tend to interpret ambiguous comments negatively or be emotionally overwhelmed by critiques. Mental stability is the other important mediator, as individuals with less stable mentality are more likely to perceive their body images negatively and display lower levels of life satisfaction (Ali et al., 2010).

Discrepancies between one’s own BMI and mainstream norms of the body weight/size might lead to stress among adolescents, though girls and boys are likely to feel stressed for different reasons. For girls, given the widely accepted endorsement for a slender body shape in Western countries, weight gaining is one of the most common concerns that teen girls have about their bodies. Overweight teen girls tend to hold negative self-perceptions (Forste and Moore, 2012), which in turn, give rise to their feelings of unworthiness, depression and low self-esteem (Cohane and Pope, 2001; Sarlio-Lähteenkorva et al., 2003; Needham and Crosnoe, 2005; Frederick et al., 2007; Merten et al., 2008; Mäkinen et al., 2012). A skinny body shape is not endorsed by boys, however. Quite the opposite, being underweight may imply the lack of strength and masculinity, and is thus more likely to be associated with negative self-perceptions and low self-esteem among boys. The question is therefore whether or not the relationship between body weight/size and life satisfaction would remain for each of the gender groups, once psychological factors are taken into account.

### Ethno-Racial Variations in Gendered Normative Perceptions

Due to the fact that perceptions of an acceptable body size differ across societies, the gender-specific association between BMI and life satisfaction is predicted to vary among youths from various ethnic and racial backgrounds (Sarlio-Lähteenkorva et al., 2003; Swami et al., 2010). For instance, obesity is stigmatized in the West, where thinness is prevalently associated with attractiveness and success, particularly among girls (Forste and Moore, 2012). Heavier bodies, on the other hand, are favoured in socioeconomically less developed societies, since there, heavyset bodies are closely associated with fertility and sexuality for women, whereas athleticism and strength for men (Swami et al., 2010).

That being said, the trend of globalization is, to a great extent, a process of westernization for non-Western cultures. Under influences of Western social media and popular culture, it is unsurprising that adolescents in non-Western societies might embrace Westernized beauty standards even more than their counterparts born in the Western world, if their normative perceptions are driven by westernization. In their comprehensive review of existing literature, Sarlio-Lähteenkorva et al. (2003) point out that Russian adolescents appear to be more dissatisfied with their body weight, despite the fact that the percentage of Russian adolescents reporting the normal weight is much higher than that of their American counterparts. In the same study, the authors also compare teenagers between Finland and Estonia, and findings show that fewer girls aged 15–16 are satisfied with their body weight in Estonia than in Finland, whereas the situation is the opposite for Finnish and Estonian boys. The authors argue that thinness in the Eastern European context could be interpreted as being positively associated with the chance of obtaining better jobs and achieving higher social status for young women, and therefore becomes the core attribute of the female body ideals among adolescent girls (Sarlio-Lähteenkorva et al., 2003).

Furthermore, the ethno-racial variation in the association between body weight and life satisfaction has been reported to be more pronounced among women (Kronenfeld et al., 2010; Swami et al., 2010). According to the meta-analysis by Grabe and Hyde (2006), black women tend to adopt an ideal of a larger and stronger body, so that they are more open to accept overweight body size, experience less social pressure about weight, and are overall more satisfied with their body images than white women. Unlike African American women, Asian American females are more likely to value the mainstream beauty standards and are hence not different from white women in terms of body satisfaction. Wardle et al. (2006) study based on a sample of university students from 22 countries worldwide reveals substantial concerns about being overweight among both Asian women and men, even though Asian students are on average slimmer than students from other ethnic/racial origins.

### The Current Study

Built upon existing literature, this study aims to address the following questions: 1) How do teen boys and girls perceive and feel about their body weights, respectively? 2) How do social and psychological factors mediate the relationship between body weight/size and life satisfaction for teen boys and girls, respectively? And 3) to what extent can the variation in youth’s life satisfaction be attributed to their ethno-racial origins? By focusing on youth in three Western European immigrant-receiving societies, this study supplement the existing literature that was mainly concentrated on the American context.

## Data and Variables

Our study employs data from the first wave of the CILS4EU (Children of Immigrants Longitudinal Survey in Four European Union Countries) study carried out among 14–15-year-old youth with and without migration background in Germany, the Netherlands and Sweden (Kalter et al., 2016; Kalter et al., 2019). Although England was also included in the CILS4EU data, it was removed from analyses due to very high non-response rates on body weight and height, information that is crucial to compute the BMI. The first wave of the CILS4EU data was collected between the end of 2010 and the beginning of 2011. Students were selected through a three-stage sampling design. First-stage sampling units were schools having students in targeted age groups, through the proportional sampling method based on school size. Prior to sampling, schools were assigned to explicit strata according to the proportion of students with immigrant backgrounds so that oversampling schools with large shares of immigrants could be possible. The second-stage units were classes within targeted grades in sampled schools, from which two classes were randomly sampled. Finally, the third-stage sampling units were students within classes. All students of a class were selected following the whole sampling method. As a result, 5,013 students from 271 classes completed questionnaires in Germany, 4,363 students from 222 classes in the Netherlands, and 5,025 students from 251 classes in Sweden. Response rates at the school level (after equivalent replacements of non-responding schools) were 90% in Germany, 69% in the Netherlands and 77% in Sweden. Response rates at the student level were 81% in Germany, 91% in the Netherlands and 86% in Sweden.^1^

The dependent variable in our analyses is life satisfaction, captured by a ten-point scale with higher values indicating higher levels of life satisfaction. The main independent variable is the classification of body mass based on BMI—body weight in kilograms divided by square of height in metres.^2^ BMI was collapsed into three categories—underweight, normal weight and overweight—based on the WHO (World Health Association) scale adjusted for adolescents’ age (in months) and gender.^3^ Among respondents who reported weight and height information,^4^ about 2.7% of all respondents in the sample are underweight, 81% are within the range of a normal weight, and almost 17% are overweight. The proportion of overweight is highest in Germany (about 20%), closely followed by Sweden (18%), and the Netherlands has the lowest proportion (10%).

As aforementioned, boys and girls hold distinctively different perceptions of what an ideal boy/girl body image should be like. Their (dis)satisfaction with their own bodies is based on comparisons with the standard, as well as other counterparts, in their own gender group. In other words, the focus of this study is the distinctive pattern of the BMI-LS (life satisfaction) relationship within each of the gender groups. Cross-gender comparisons on the BMI-LS association would be unwarranted, due to different body weight/size standards imposed on boys and girls. For this reason, we carry out analyses for boys and girls separately.^5^

The variation of life satisfaction is sufficient enough to be treated as a continuous variable, suitable for Ordinary Least Square (OLS) regression models. We have also run Ordinal Logistic Regression models by collapsing the ten-point scale of life satisfaction into three groups, and find patterns in line with those reported by OLS models. Detailed information is presented in the Sensitivity Analysis section.

Other variables pertaining to respondents’ demographic characteristics are age, groups of origin and immigrant generations. Groups of origin are a series of dummy variables, including the majority native-born population (used as a reference group), referring to individuals who themselves and whose parents were born in the survey country. Among those who reported migration background, we differentiate young people originating from Western Europe, Eastern Europe, Sub-Saharan Africa or the Caribbean, Middle East and Northern Africa (MENA), and the rest of Asia, based on the information on their own countries of birth as well as their parents’ countries of origin.^6^ Individuals originating from other countries and those from unknown groups of origin are categorized as “others.”

We control for generational status, including 1) the first generation, i.e., young people who migrated themselves, 2) the second generation, i.e., children of immigrants, and 3) children from mixed parental background. For the third group we differentiate between children of transnational marriages (with one of the respondent’s parents having direct migration experience whereas another being native-born but having at least one immigrant parent) and children of intermarriages (with one of the parents being a migrant her/himself whereas another being native-born and having no immigrant parent). Controlling for migration status is necessary, for the purpose of tracing possible differences related to one’s own experience of migration^7^ and the contexts of family socialisation.

In addition to demographic characteristics, we further control for respondents’ family type, which is a dummy variable indicating whether or not a child grew up in a single-parent household, and family background measured by the highest level of parental educational attainment. A set of dummy variables captures a range of educational levels for parents, varying from primary education or none (used as the reference category) to lower secondary, to upper secondary, and finally to tertiary education. Respondents with missing information on parental origin form a separate group.

The first model, in which only socio-demographic characteristics are included, serves as the benchmark model. Based on it, we first explore the importance of social relationships in mediating the association between body weight and life satisfaction, by focusing on the roles of parents, teachers and peers.^8^ The parent–child relationship is captured by a mean score of three items, including whether parents try to comfort a child when s/he is sad, whether parents often criticize a child, and whether parents try to understand what a child thinks or how s/he feels. Higher values of this variable, measured by a five-point scale, refer to more comforting parent-child relations. The variable “teacher–child relation” is captured by a mean score of two items “teachers encourage me at school,” and “there are teachers who treat me unfairly,” which are recoded so as to vary in the same direction in a five-point scale. Higher values of the generated variable refer to more positive teacher–child relations. The variable “peer relation” is measured by a mean score pertaining to questions of whether the respondent is teased or bullied at school. Answers to the questions on how often a respondent is teased or bullied by other students in the month prior to the interview time are coded as a four-point scale, namely, every day, once or several times a week, less often than once a week, and never. Similar to other variables, we code the peer relation variable in the order for higher values to reflect more favourable peer relations.

The second set of focal mediators are psychological factors. One is self-esteem, derived from a set of three questions capturing whether the person has a lot of good qualities, has a lot to be proud of, likes her/himself the way s/he is. Responses to the three questions range on a five-point scale from strongly agree to strongly disagree. The generated variable represents a mean score of the three variables and is coded with higher values standing for more positive perceptions of oneself. Another variable, mental state is a mean score of two items measuring feelings of depression and unworthiness. Both variables range on a four-point scale (including the categories “often true,” “sometimes true,” “rarely true,” and “never true”), and the generated variable is the mean score of the two items with higher values representing better mental well-being.

## Descriptive Patterns

Table 1 reports two parts of descriptive statistics, frequency distributions by regions of origin, as well as means and standard deviations of major mediating variables. Both parts of descriptive information are reported by BMI groups and for boys and girls separately. In terms of sample distributions among the three BMI groups, boys and girls have virtually the same proportion of being underweight, but the percentage of girls being overweight is only a half of that of their boy counterparts. Taking a close look at the distribution by regions of origin within each BMI group, one can clearly see that chances for respondents from certain regions falling into certain BMI groups are by no means random. Among boys, those originating from Sub-Saharan Africa/the Caribbean show a percentage of being underweight that doubles their percentage in the overweight group. The similar pattern is also observed among Asian boys, with a slightly smaller gap between those two percentages. By contrast, those originating from the MENA area present a percentage of being overweight that is almost double their percentage of being underweight. Among girls, those originating from the MENA area are also more often found in the overweight group (their percentage in the overweight group almost doubles that in the underweight group), whereas Asian girls are very likely to be underweight (their percentage in the underweight group is more than triple their percentage in the overweight group). We must point out that when the gender subsamples are further divided by ethnic origin, the size of the “underweight” group becomes considerably small for both boys and girls, as shown by Table 1. This we consider one of the major limitations of this paper. In the robustness check shown in the Supplementary Appendix SA, we therefore regroup the BMI distribution by differentiating the interquartile range (25th–75th percentile) from the lowest and highest 25th percentile, and name them as “normal weight,” “underweight” and “overweight,” respectively. The major finding based on this alternative grouping method remains consistent with what is presented in this paper. More details are discussed in the following section.

**TABLE 1:** Sample distributions, means and standard deviations (in parentheses) for major mediating variables.

	Underweight		Normal weight		Overweight		All	
Boys	143	2.0%	5,425	75.4%	1,624	22.6%	7,192	100.0%
Ethnic origin								
Native born	89	62.2%	3,325	61.3%	842	51.8%	4,256	59.2%
Western Europe	5	3.5%	283	5.2%	105	6.5%	393	5.5%
Eastern Europe	13	9.1%	425	7.8%	166	10.2%	604	8.4%
Sub-Saharan Africa and Caribbean	10	7.0%	255	4.7%	53	3.3%	318	4.4%
Middle East and Northern Africa (MENA)	20	14.0%	897	16.5%	390	24.0%	1,307	18.2%
Asia	6	4.2%	140	2.6%	46	2.8%	192	2.7%
Others	0	0.0%	100	1.8%	22	1.4%	122	1.7%
Social factors								
Parent-child relations (range 1–5)	3.99	(0.61)	3.93	(0.71)	3.81	(0.77)	3.92	(0.72)
Teacher-child relations (1–5)	3.22	(0.81)	3.28	(0.92)	3.22	(0.94)	3.26	(0.94)
Peer relations (1–4)	3.56	(0.60)	3.73	(0.52)	3.67	(0.58)	3.71	(0.54)
Psychological factors								
Self-esteem (1–5)	4.01	(0.57)	4.13	(0.57)	4.09	(0.63)	4.11	(0.60)
Mental state (1–4)	3.15	(0.70)	3.38	(0.63)	3.27	(0.71)	3.34	(0.66)
Girls	134	1.9%	6,222	86.4%	848	11.8%	7,204	100.0%
Ethnic origin								
Native born	90	67.2%	3,681	59.2%	471	55.5%	4,242	58.9%
Western Europe	8	6.0%	333	5.4%	45	5.3%	386	5.4%
Eastern Europe	7	5.2%	545	8.8%	69	8.1%	621	8.6%
Sub-Saharan Africa and Caribbean	4	3.0%	296	4.8%	42	5.0%	342	4.7%
Middle East and Northern Africa	17	12.7%	1,071	17.2%	195	23.0%	1,283	17.8%
Asia	7	5.2%	188	3.0%	13	1.5%	208	2.9%
Others	1	0.7%	108	1.7%	13	1.5%	122	1.7%
Social factors								
Parent-child relations (1–5)	3.90	(0.88)	3.93	(0.81)	3.89	(0.85)	3.92	(0.82)
Teacher-child relations (1–5)	3.38	(0.87)	3.32	(0.85)	3.38	(0.88)	3.34	(0.86)
Peer relations (1–4)	3.70	(0.56)	3.74	(0.50)	3.59	(0.66)	3.72	(0.53)
Psychological factors								
Self-esteem (1–5)	3.88	(0.67)	3.85	(0.67)	3.72	(0.74)	3.83	(0.69)
Mental state (1–4)	3.01	(0.81)	2.97	(0.78)	2.83	(0.86)	2.96	(0.79)

Sources: CILS4EU, wave 1, weighted data, authors’ calculations.

1 Higher values pertain to more positive/favourable characteristic on each of the respective variables.

2 Row percentages are reported for “Boys” and “Girls.” column percentages are reported for ethnic origin breakdown.

Regarding major mediating variables, boys with normal weight enjoy the most favourable relations with their teachers and peers, have the highest level of self-esteem and are the least depressive. However, it is underweight boys who report the most comforting relations with their parents, though the average quality of relations with parents does not fall far behind for normal-weight boys. For girls, qualities of social relations with parents and teachers do not seem to differ drastically across three BMI groups. However, with respect to peer relations, overweight girls report much worse relations with peers, compared to their underweight and normal-weight counterparts. Psychological traits vary with girls’ body weight, with both self-esteem and mental health descending from the underweight to normal-weight, and then to overweight group.

**FIGURE 1:**
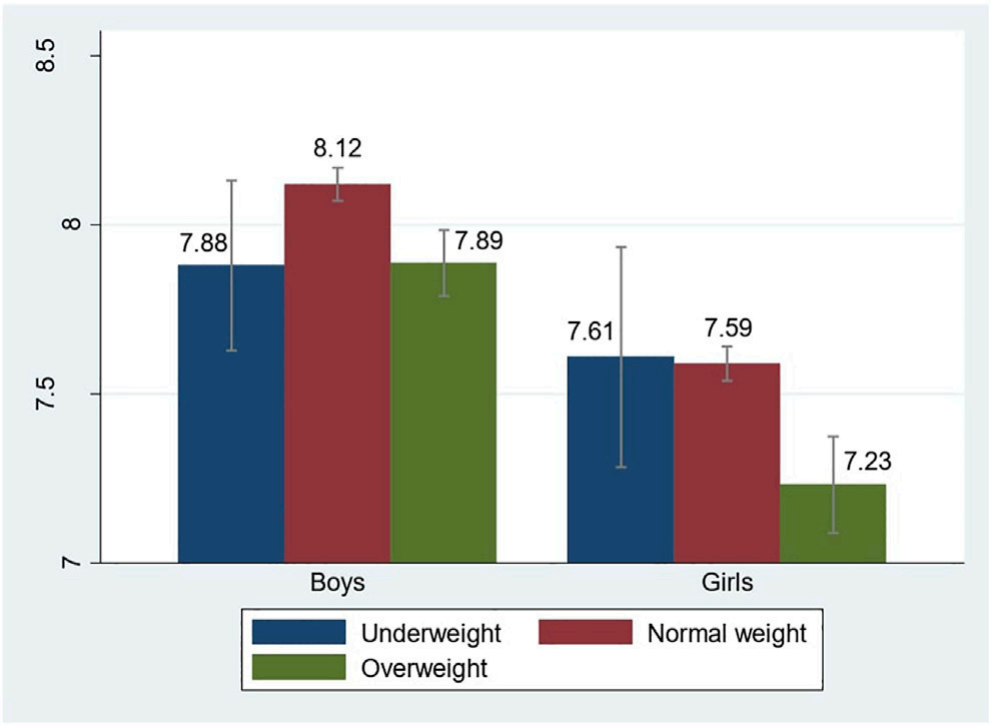
Life satisfaction by body mass index and gender.

Figure 1 subsequently presents whether the patterns of life satisfaction by BMI among Western European teen boys and girls are similar to those reported elsewhere. For male adolescents, we observe an inverse U-shape pattern, echoing the findings of earlier research (Mäkinen et al., 2012; Linna et al., 2013). Girls are evidently dissatisfied when they fall into the overweight range, but the life satisfaction gap between being normal weight and underweight is too trivial to be significant. This trend is not completely in line with existing research, which indicates a linear relationship between weight and life satisfaction among girls (Pingitore et al., 1997; Mäkinen et al., 2012). In accordance with the widely documented gender gap in life satisfaction, life satisfaction in general is much lower among girls than among boys in our data.

## Multivariate Analyses

In all models presented in this section, we control for fixed effects at the level of survey countries (Germany used as a reference category).^9^ All model estimations are processed by using analytical weighting. A single weight variable, which combines multi-stratum sampling weights, adjustments for non-responses rates, as well as rescaling to reflect the actual sample size, is used for this purpose (see more details in the CILS4EU technical report^10^). Missing values are deleted pairwise, as different models include different sets of variables.

In Table 2, we explain the BMI-LS association by establishing the relative importance of social and psychological mediating factors. The baseline model (Model 1) includes respondents’ socio-demographic characteristics, such as age, ethnic origin, migration status, social background and country of residence. We just point out that each model is run twice, for boys and girls respectively, due to the cross-gender incomparability regarding the BMI-LS association, as aforementioned. Model 1 confirms the aforementioned inverse U-shaped pattern with both underweight and overweight boys being less satisfied with their lives compared to their normal-weight counterparts, though the coefficient is only significant for overweight boys. The same significant pattern is observed among girls. Also the gap of life satisfaction between overweight and normal weight girls is larger than that between overweight and normal weight boys.

**TABLE 2:** Coefficients of body weight across OLS regressions on life satisfaction by gender in Germany, Sweden and the Netherlands.

	Boys (normal weight—ref.)							Girls (normal weight—ref.)						
	Underweight		Overweight		Obs #	R^2^		Underweight		Overweight		Obs #	R^2^	
	Coef.	SE^a^	Coef.	SE^a^		Cross-model comparison (*p*)^b^		Coef.	SE^a^	Coef.	SE^a^	—	Cross-model comparison (*p*)^b^	
Model 1 (socio-demographics)	−0.27	(0.17)	−0.21*	(0.10)	6,435	0.04	—	−0.02	(0.23)	-0.34**	(0.12)	6,378	0.03	—
Model 2 (M1 + parents)	−0.34*	(0.16)	−0.09	(0.09)	6,216	0.13	0.25	0.02	(0.21)	−0.29**	(0.10)	6,203	0.20	0.58
Model 3 (M1 + teachers)	−0.26	(0.18)	−0.20+	(0.10)	6,418	0.05	0.87	−0.05	(0.22)	−0.35**	(0.12)	6,371	0.06	0.53
Model 4 (M1 + peers)	−0.21	(0.16)	−0.17+	(0.10)	6,422	0.07	0.14	0.01	(0.24)	−0.23+	(0.12)	6,364	0.05	0.00
Model 5 (M1 + all social factors)	−0.29^+^	(0.16)	−0.07	(0.09)	6,190	0.16	0.95	0.02	(0.21)	−0.23*	(0.11)	6,184	0.22	0.10
Model 6 (M1 + self-esteem)	−0.20	(0.17)	−0.10	(0.11)	6,425	0.14	0.58	−0.15	(0.19)	−0.07	(0.12)	6,370	0.18	0.00
Model 7 (M1 + mental state)	−0.06	(0.18)	−0.12	(0.11)	6,420	0.15	0.03	−0.06	(0.21)	−0.17	(0.11)	6,373	0.28	0.01
Model 8 (M1 + all psychological factors)	−0.06	(0.18)	−0.06	(0.11)	6,410	0.20	0.09	−0.12	(0.19)	−0.07	(0.11)	6,366	0.31	0.00
Model 9 (M1 + social + psychological)	−0.14	(0.15)	−0.01	(0.10)	6,173	0.24	0.06	−0.07	(0.19)	−0.07	(0.10)	6,174	0.35	0.00

Sources: CILS4EU, wave 1, weighted data, authors’ calculations.

**p < 0.01, *p < 0.05, + *p*< 0.10. The following variables are also controlled in the models: survey country, age, origin groups, generational status, parental education, and type of household.

a Standard errors, presented in the second column, are in parenthesis.

b The level of significance (shown by the *p* value) of the change in respective coefficients for the dummy-coded variable “underweight” and “overweight” (“normal weight” as the reference category) in Models 2–9 compared to Model 1 is indicated in the column of “cross-model comparison (p)”.

From Models 2 through 4, the variables capturing social relations are taken into account. Comparing Models 2 and 1, one can see that the inclusion of relations with parents changes the patterns of the BMI-LS association for both underweight and overweight boys. With the quality of relations with parents, as well as other covariates, being equal, underweight boys are, on average, significantly less satisfied than normal-weight boys with their lives, whereas the negative association between being overweight and life satisfaction is no longer significant. This means that among boys, those underweight ones actually run a higher risk of feeling unsatisfied than their overweight counterparts. This difference is disguised, because underweight boys might receive more protection and support from their parents whereas overweight boys might feel more pressure from their parents. Models 3 and 4 show that controlling for relations with teachers and peers does not change the level of life satisfaction for underweight boys significantly, but does make the lower level of life satisfaction only marginally significant for overweight boys. This means that those relationships do not matter significantly in mediating the BMI-LS association for underweight boys, whereas they do matter to a certain degree for overweight boys. Among girls, controlling for social relations does not change the patterns of association between life satisfaction and body weight for both underweight and overweight girls. A single exception is that relations with peers seem to attenuate the strength of association between body weight and life satisfaction for overweight girls.

Model 5 controls for social relations in all three respects. It shows that after qualities of social relations with parents, teachers and peers, as well as other covariates were controlled, underweight boys indeed feel less satisfied than their normal-weight counterparts with their lives, only at the level of marginal significance. By contrast, overweight boys are not significantly less satisfied than normal-weight boys. Differences between Models 5 and 1 indicate that while underweight boys have real concerns about their body weight, high-quality relations with their parents play a crucial role in boosting their life satisfaction. On the contrary, while overweight boys do not really concern about their body weight itself, poor qualities of their social relations, particularly with parents, drive their dissatisfaction with lives. Patterns for girls are different. Social relations do not seem to matter for life satisfaction of underweight girls. For overweight girls, the inclusion of peer relations reduces the gap of life satisfaction relative to normal-weight girls the most (from −0.34 to −0.23). The second important mediator is their relations with parents. However, life satisfaction of overweight girls remains significantly lower than their normal-weight counterparts, with all aspects of social relations taken into account.

From Models 6 to 8, psychological factors are included one by one and then together. Results show that controlling for psychological factors changes the effect sizes and the levels of significance of BMI coefficients for both overweight boys and girls. At same levels of self-esteem and mental stability and with other covariates being equal, overweight youth are no longer significantly less satisfied with their lives than their normal weight counterparts. This implies that overweight youth’s low life satisfaction essentially reflects self-perceptions driven by their low levels of self-esteem and mental stability. In the final model (Model 9), we account for both social relations and psychological factors, and it does not change the established patterns shown in Model 8.

To summarize, with only socio-demographic traits taken into account, patterns of the BMI-LS relationship are closely in line with those presented in Figure 1 for both boys and girls. Between the psychological and social factors, the former aspect seems to play a more important role, as accounting for psychological factors changes the pattern of BMI-LS association substantially for both boys and girls. This can be seen from cross-model ttest results, which show significant differences in BMI coefficients before and after the inclusion of psychological factors. Social relations seem to operate for underweight and overweight boys in opposite ways, though results of the t-test across models suggest that the inverse U-shaped pattern of the BMI-LS association remains among boys, whether social relations are controlled or not. For girls, the inclusion of peer relations reduces the negative BMI-LS association significantly in the overweight group, when we compare BMI coefficients between Models 4 and 1 (*p* = 0.00). Even after accounting for all types of social relations, life satisfaction of overweight girls remains significantly lower.

Building on the literature about normative perceptions, we further examine whether the BMI–LS association also varies with youth’s migration background. We have run analyses for both gender groups, and noteworthy patterns are only found among girls, as presented by Table 3. Other covariates being equal, overweight girls report significantly lower life satisfaction than their normal weight counterparts. This significant difference remains when social relations are taken into account, but disappears once psychological factors are included. Those findings are consistent with what Table 2 presents. More nuanced patterns are revealed by Table 3, however. The inclusion of psychological factors is associated with a significant regional difference, with girls originating from MENA and Asia being less satisfied with their lives compared to their native-born counterparts, though the lower life satisfaction of MENA girls is only marginally significant after social relations are also included.

**TABLE 3:** Coefficients of body weight across OLS regressions on life satisfaction among adolescent girls in Germany, Sweden and the Netherlands, interactions with ethnic origin.

	Model 10 (socio-demographics)		Model 11 (M1 + social factors)		Model 12 (M1 + psychological factors)		Model 13 (M1 + social + psychological factors)	
Underweight	−0.08	(0.25)	−0.03	(0.25)	−0.12	(0.23)	−0.07	(0.24)
Overweight	−0.31*	(0.14)	−0.26*	(0.12)	−0.04	(0.13)	−0.07	(0.12)
Western Europe	0.25	(0.21)	0.03	(0.19)	-0.11	(0.17)	-0.15	(0.16)
Eastern Europe	0.17	(0.18)	0.15	(0.14)	-0.20	(0.14)	−0.11	(0.13)
Sub-Saharan Africa and Caribbean	0.26	(0.21)	0.25	(0.20)	−0.27	(0.23)	-0.19	(0.19)
MENA	0.10	(0.15)	0.06	(0.13)	−0.28*	(0.12)	−0.22+	(0.13)
Asia	−0.16	(0.20)	-0.02	(0.18)	−0.45**	(0.17)	−0.31*	(0.17)
Underweight *								
Western Europe	−0.18	(0.64)	0.11	(0.50)	−0.30	(0.65)	0.13	(0.58)
Eastern Europe	0.90*	(0.40)	0.69*	(0.35)	0.31	(0.40)	0.32	(0.36)
Sub-Saharan Africa and Caribbean	−0.46	(0.48)	−0.88*	(0.40)	−1.00+	(0.56)	−1.14*	(0.49)
MENA	−0.26	(0.78)	0.00	(0.67)	−0.22	(0.67)	−0.07	(0.55)
Asia	1.27	(1.24)	0.65	(0.74)	0.51	(0.78)	0.31	(0.61)
Overweight *								
Western Europe	0.06	(0.39)	0.40	(0.28)	0.07	(0.27)	0.25	(0.27)
Eastern Europe	−0.16	(0.36)	0.41	(0.32)	−0.01	(0.29)	0.21	(0.29)
Sub-Saharan Africa and Caribbean	−0.58	(0.43)	−0.36	(0.40)	−0.43	(0.53)	−0.28	(0.50)
MENA	−0.07	(0.43)	−0.01	(0.35)	−0.22	(0.35)	−0.14	(0.33)
Asia	−0.64	(1.65)	−0.89	(1.77)	−0.06	(1.21)	−0.31	(1.37)
Obs #	6,378	—	6,184	—	6,366	—	6,174	—
R^2^	0.03	—	0.22	—	0.31	—	0.35	—

Sources: CILS4EU, wave 1, weighted data, authors’ calculations.

*p < 0.05;+< 0.10. The following variables are also controlled in the models: survey country, age, origin groups, generational status, parental education, and type of household.

There are also regional differences in girls’ perceptions of being underweight, but not of being overweight. Compared to native-born girls, being underweight is associated with a higher level of life satisfaction among girls originating from Eastern Europe, though such a difference becomes non-significant once psychological factors are taken into account. Substantially lower levels of life satisfaction are found among underweight girls from Sub-Saharan Africa and the Caribbean, with all covariates being equal. This implies: 1) that girls originating from Sub-Saharan Africa and the Caribbean may carry norms of beauty somewhat different from those in mainstream Western societies, as they do not perceive an overly slim body as the ideal body image; and 2) that social and psychological factors both play significant buffering roles in alleviating the negative perceptions of being underweight among teen girls with roots in this area. In Supplementary Table S2 of the Appendix, we have conducted the robustness check by using the alternative grouping method for the three BMI groups, and our major findings remain consistent (about lower levels of life satisfaction among girls originating from MENA and Asian areas, and more importantly, about lower life satisfaction of underweight girls from Sub-Saharan African and Caribbean areas). Nevertheless, coefficients for the main effect of being overweight and for underweight girls originating from Eastern Europe are no longer significant. Extra caution is therefore required when researchers generalize ethno-racially specific results shown by Table 3.

## Sensitivity Analysis

In addition to examining life satisfaction as a continuous variable, we have also collapsed ten points of life satisfaction into three groups, namely, “unsatisfied” including everyone who reported a life satisfaction score lower than 8, “satisfied” referring to respondents reporting the median value of life satisfaction (8), and “very satisfied” including all respondents reporting life satisfaction scores higher than 8. We subsequently ran the Ordinal Logistic Regression modelling on the new three-group “life satisfaction” variable. Results strongly resonate with those presented in Table 2.

As Table 4 shows, among boys, the BMI-LS association shown in Model 14 follows an inverse U-shaped pattern. Social relations play opposite roles in mediating the BMI-LS association among underweight and overweight boys. A comparison between Models 18 and 14 reveal that accounting for social relations enhances the negative BMI-LS association for underweight boys but decreases that association for overweight boys to the level of non-significance. This suggests, as aforementioned, that social relations play a positive role in boosting underweight boys’ life satisfaction. As for overweight boys, they would not have felt dissatisfied if it were not for poor qualities of social relations they experience.

**TABLE 4:** Ordinal Logistic Regression models on life satisfaction by gender in Germany, Sweden and the Netherlands.

	Boys (normal weight—ref.)						Girls (normal weight—ref.)					
	Underweight		Overweight		Ob#	Log pseudolikelihood	Underweight		Overweight		Ob#	Log pseudolikelihood
	Coef.	SE	Coef.	SE			Coef.	SE	Coef.	SE		
Model 14 (socio-demographics)	−0.40*	(0.20)	−0.21*	(0.10)	6,435	−6,798	0.03	(0.22)	−0.32**	(0.11)	6,378	−6,938
Model 15 (M1 + parents)	−0.59**	(0.19)	−0.08	(0.09)	6,216	-6,154	0.06	(0.24)	−0.32**	(0.11)	6,203	−6,084
Model 16 (M1 + teachers)	−0.41*	(0.19)	−0.21*	(0.10)	6,418	−6,687	−0.00	(0.23)	−0.35**	(0.12)	6,371	−6,809
Model 17 (M1 + peers)	−0.33+	(0.19)	−0.17+	(0.10)	6,422	−6,684	0.09	(0.23)	−0.20+	(0.11)	6,364	−6,800
Model 18 (M1 + all social factors)	−0.52**	(0.18)	−0.05	(0.09)	6,190	−6,012	0.10	(0.23)	−0.26*	(0.12)	6,184	−5,974
Model 19 (M1 + self-esteem)	−0.31	(0.24)	−0.08	(0.11)	6,425	−6,323	−0.13	(0.23)	−0.10	(0.13)	6,370	−6,417
Model 20 (M1 + mental state)	−0.09	(0.25)	−0.10	(0.11)	6,420	−6,349	0.08	(0.22)	−0.23+	(0.12)	6,373	−6,016
Model 21 (M1 + all psychological factors)	−0.07	(0.28)	−0.02	(0.12)	6,410	−6,061	-0.03	(0.23)	−0.12	(0.13)	6,366	−5,878
Model 22 (M1 + social + psychological)	−0.25	(0.23)	0.06	(0.11)	6,173	−5,610	0.02	(0.23)	−0.11	(0.13)	6,174	−5,446

Sources: CILS4EU, wave 1, weighted data, authors’ calculations.

**p < 0.01, *p < 0.05, + *p*< 0.10. The following variables are also controlled in the models: survey country, age, origin groups, generational status, parental education, and type of household. Standard errors, presented in the second column, are in parenthesis.

Among girls, confirming results presented above, Model 14 shows that overweight girls are less satisfied than their normal weight counterparts, whereas there is no significant difference in life satisfaction between underweight and normal weight girls. The comparison between Models 18 and 14 reveals that the life satisfaction gap between overweight and normal weight girls remains even with social relations taken into account, though the inclusion of social relations does account for the life satisfaction gap to some extent.

From Models 19 through 22, the inclusion of psychological traits turns the BMI-LS associations to non-significant for both boys and girls. This confirms the essential role psychological traits play in differentiating youth’s self-perceptions of body weight, which in turn, is associated with different levels of life satisfaction.

## Summary and Conclusion

The period of adolescence is marked by numerous challenges, due to physiological and psychological transitions, as well as life events typically occurring at this stage, such as dating and changing relationships with parents. Coping with all the challenging situations might leave imprints on young people’s subjective well-being. In this study, we examined one specific aspect, the relationship between adolescents’ body weight/size and life satisfaction.

Our findings have shown that patterns of the BMI–LS association observed in the US and other contexts also hold true for adolescents in three European countries—Germany, the Netherlands and Sweden. There is an inverse U-shaped relationship between boys’ BMI and life satisfaction, though the life satisfaction gap is more salient between underweight and normal weight boys, and non-significant or only marginally significant between overweight boys and their normal weight counterparts. Moreover, the association between being underweight and life satisfaction among girls varies across ethno-racial groups. Girls originating from Eastern Europe have a tendency to gain more life satisfaction when being underweight, whereas girls rooted in Sub-Saharan African and Caribbean countries display consistently low levels of life satisfaction when being underweight. Those findings well reflect the normative nature of body weight/size perceptions. By and large, both native-born and immigrant youth’s perceptions of their bodies are dominantly shaped by the cultural norm cultivated in Western countries, which endorses a strong male body and a slender female body as ideal body images (except for those originating from Sub-Saharan Africa and the Caribbean).

As aforementioned, one of the major limitations of this study is the small sizes of underweight groups for both boys and girls, which imposes particular challenges when the BMI-LS association further intertwines with ethnic origin. Fortunately, the use of the alternative grouping methods for the three BMI categories (in the Supplementary Table S2) proves the robustness of major findings presented by our main analyses.

We want to point out that BMI does not differentiate between fat and muscle tissue (Cohane and Pope, 2001). It is likely that BMI in the overweight range is due to excessive muscles rather than fat, particularly among boys. This might explain why no significantly negative association is found between being overweight and life satisfaction among boys. In addition, physical changes teen boys go through are likely to lead to lean body shapes (Linna et al., 2013), corresponding to our finding on boys’ discontent about being underweight rather than overweight.

In terms of social mediating factors, relationships with parents (particularly among boys) and relationships with peers (particularly among girls) play significant roles. Those findings suggest the importance of social policies on enhancing the parent-child relation in the domestic arena as well as combatting bully behaviour in the public arena in improving subjective well-being of adolescents, particularly who have weight concerns. Psychological factors, captured by mean scores of items related to self-esteem and mental state, explain the association between BMI and life dissatisfaction substantially, for both boys and girls. Due to its cross-sectional nature, our study is unable to solve the causal puzzle of whether individuals with low self-esteem, depressive state or low subjective well-being tend to develop weight-related problems or, reversely, whether societally “unacceptable” weight conditions lead to lower self-esteem, depressions or lower life satisfaction (Markowitz et al., 2008). Future research utilizing panel data sets on this topic is very much needed. Regardless, such finding highlights that youth’s self-perceptions of their bodies are inherently associate with their levels of self-esteem and mental stability. Namely, being either overweight or underweight does not necessarily hurt youth’s life satisfaction, as long as they do not internalize the “standard” advocated by social media and popular culture. Policies and other resources, which target at strengthening youth’s self-confidence, cultivating their awareness of self-worthiness and sustaining their mental health at multiple levels (such as home, school and society), are thus of great importance.

The self-reporting of BMI is prone to criticism as well (Villanueva, 2001; Kronenfeld et al., 2010). Research shows that adults tend to under-report their weight and over-report their height (Elgar et al., 2005; Wardle et al., 2006; Brunner Huber, 2007; Sherry et al., 2007). Girls seem to be more likely to under-report weight than boys (Sherry et al., 2007). However, our findings about girls reveal that the significant group difference in the BMI-LS association only exists between those who are overweight and those who are not. Namely, if systematic biases existed in our estimations, those biases would only make coefficients harder to be significant, supporting the robustness of those patterns presented in the text.

Above all, this study demonstrates the existence of significant relationships between body weight/size and life satisfaction, using data beyond the American context. It also sheds light on the importance of mediating factors in the BMI-LS association, including social and psychological factors as well as ethno-racial diversity. Future research is much needed to deepen scholarly understanding about the formation of normative perceptions of body shape; for example, to reveal the unknown roles of social media and popular culture in internalizing the westernized body weight/size norm, to explore the interplay between external social influences and internal psychological factors in shaping youth’s normative perceptions, and to further detangle how the mediating roles played by all those factors differ across ethno-racial groups. We hope findings from the current study serve as a stepping-stone to advance this line of research.

## Data Availability

The datasets presented in this study can be found in online repositories. The names of the repository/repositories and accession number(s) can be found below: https://www.cils4.eu/.

## Footnotes

1 For more details on the study design and technical issues, see Kalter et al. (2019) and www.cils4.eu.

2 Both weight and height are self-reported.

3 Grouping thresholds are retrieved from https://www.who.int/tools/growth-reference-data-for-5to19-years/indicators/bmi-for-age.

4 Five cases were excluded because of missing information on either weight or height.

5 We have also run interactions with two gender groups included in the same model, and the results confirm that the life satisfaction gap between boys and girls is almost completely associated with the gender factor itself (results are not presented, but available upon request).

6 For respondents whose parents were born in the same region, their ethnic background was coded in line with their parents’ birth region, regardless of their own birth country. For respondents whose parents were born in different regions, there were two scenarios. One is that the respondent was born in the same region with one of the parents, in which case his/her ethnic background was coded based on his/her own birth country. The other is that the respondent and two parents were born in three different regions. If so, the respondent’s ethnic background was coded in line with the country origin of the head of the household, which referred to the father’s birth place in two-parent families and the birth place of the parent with whom the respondent mainly lived in single-parent families. In complicated scenarios, however, information about birth countries of either the respondents themselves or one or both of their parents was often missing, so that those cases were treated as missing values and excluded from analyses (such cases constituted about 1% of the total sample).

7 To participate in the study, the respondents had to be sufficiently fluent in the host-country languages. Unsurprisingly very few first-generation migrants are recent arrivals.

8 For the original wording of the questions consult the following website: http://www.cils4.eu/images/wave1_material/master/w1_ym_master.pdf.

9 Due to reduced sample sizes we do not carry separate analyses by countries.

10
https://www.cils4.eu/images/wave1_material/technical/za5353_technicalreport_wave1.pdf
