# Supplementary material for: Gendered Body Mass and Life Satisfaction Among Youth in Three Western European Immigrant-Receiving Countries
Source: Front Sociol. 2021 Dec 10;6:695374. doi: 10.3389/fsoc.2021.695374 (PMC8705931; doi:10.3389/fsoc.2021.695374)
Supplement: Supplementary file 1 [file Table1.pdf]

Appendix

Table A1: Means and standard deviations (in parentheses) for major mediating variables

	Underweight		Normal weight		Overweight		All	
Boys	1507	22.9%	3217	48.8%	1870	28.4%	6594	100%
Ethnic origin								
Native born	998	66.2%	1967	61.1%	958	51.2%	3923	59.5%
Western Europe	60	4.0%	164	5.1%	129	6.9%	353	5.4%
Eastern Europe	98	6.5%	267	8.3%	191	10.2%	556	8.4%
Sub-Saharan Africa &	82	5.4%	131	4.1%	67	3.6%	280	4.2%
Middle East and Northern Africa (MENA)	182	12.1%	564	17.5%	447	23.9%	1193	18.1%
Asia	57	3.8%	69	2.1%	54	2.9%	180	2.7%
Others	30	2.0%	55	1.7%	24	1.3%	109	1.7%
Girls	1803	27.7%	3309	50.8%	1405	21.6%	6517	100%
Ethnic origin								
Native born	1169	64.8%	1942	58.7%	746	53.1%	3857	59.2%
Western Europe	101	5.6%	184	5.6%	76	5.4%	361	5.5%
Eastern Europe	142	7.9%	306	9.2%	125	8.9%	573	8.8%
Sub-Saharan Africa &	72	4.0%	155	4.7%	73	5.2%	300	4.6%
Middle East and Northern	228	12.6%	569	17.2%	334	23.8%	1131	17.4%
Asia	62	3.4%	99	3.0%	25	1.8%	186	2.9%
Others	29	1.6%	54	1.6%	26	1.9%	109	1.7%

Sources: CILS4EU, wave 1, weighted data, authors' calculations.

Note: 1. The three BMI groups are generated by dividing the gender-specific BMI distribution of the whole sample as the lowest 25th percentile (upper limit not included), 25th-75th percentile (upper limit not included) and 75th-100th percentile.

2. Higher values pertain to more positive/favourable characteristic on each of the respective variables.

3. Row percentages are reported for "Boys" and "Girls." Column percentages are reported for ethnic origin breakdown.
