# Supplementary material for: Gendered Body Mass and Life Satisfaction Among Youth in Three Western European Immigrant-Receiving Countries
Source: Front Sociol. 2021 Dec 10;6:695374. doi: 10.3389/fsoc.2021.695374 (PMC8705931; doi:10.3389/fsoc.2021.695374)
Supplement: Supplementary file 2 [file Table2.pdf]

Table A2: Coefficients of body weight across OLS regressions on life satisfaction among adolescent girls in Germany, Sweden and the Netherlands, interactions with ethnic origin

	Model 10 (socio-demographics)		Model 11 (M1 + social factors)		Model 12 (M1 + psychological factors)		Model 13 (M1 + social + psychological factors)	
Underweight	0.14	(0.11)	0.05	(0.11)	0.00	(0.08)	-0.01	(0.08)
Overweight	-0.19	(0.12)	-0.15	(0.11)	-0.02	(0.12)	-0.03	(0.10)
Western Europe	0.19	(0.24)	-0.01	(0.22)	-0.17	(0.19)	-0.21	(0.19)
Eastern Europe	0.23	(0.24)	0.19	(0.19)	-0.18	(0.19)	-0.08	(0.18)
Sub-Saharan Africa & Caribbean	0.48*	(0.23)	0.47+	(0.24)	0.01	(0.22)	0.10	(0.22)
MENA	0.02	(0.16)	-0.03	(0.15)	-0.38**	(0.14)	-0.30*	(0.14)
Asia	0.06	(0.23)	0.11	(0.21)	-0.42*	(0.20)	-0.29	(0.18)
Underweight *								
Western Europe	0.16	(0.33)	0.17	(0.32)	0.15	(0.30)	0.18	(0.31)
Eastern Europe	-0.31	(0.32)	-0.12	(0.27)	-0.19	(0.26)	-0.14	(0.24)
Sub-Saharan Africa & Caribbean	-0.62	(0.42)	-0.56	(0.43)	-0.92*	(0.39)	-0.80*	(0.39)
MENA	0.10	(0.37)	0.17	(0.29)	0.24	(0.23)	0.18	(0.23)
Asia	-0.20	(0.46)	-0.10	(0.35)	0.17	(0.33)	0.14	(0.29)
Overweight *								
Western Europe	0.07	(0.32)	0.24	(0.28)	0.13	(0.25)	0.22	(0.25)
Eastern Europe	0.15	(0.34)	0.28	(0.29)	0.16	(0.27)	0.16	(0.26)
Sub-Saharan Africa & Caribbean	-0.51	(0.39)	-0.44	(0.38)	-0.45	(0.43)	-0.39	(0.42)
MENA	0.18	(0.34)	0.21	(0.29)	0.03	(0.30)	0.09	(0.28)
Asia	-0.85	(1.00)	-0.77	(1.10)	-0.36	(0.73)	-0.41	(0.82)
Constant	10.11***	(1.19)	3.07**	(0.99)	5.68***	(0.81)	3.11***	(0.8)
Obs #	6378		6184		6366		6174	
R^2	0.03		0.22		0.31		0.35	

Sources: CILS4EU, wave 1, weighted data, authors' calculations.

Note: * $p < 0.05$; $^{+} < 0.10$. The following variables are also controlled in the models: survey country, age, origin groups, generational status, parental education, and type of household.
